# Effect of Different Degrees of Deep Freezing on the Quality of Snowflake Beef during Storage

**DOI:** 10.3390/foods11152175

**Published:** 2022-07-22

**Authors:** Yawei Chang, Yan Liu, Yun Bai, Shuang Teng, Yiping Guo, Han Dou, Keping Ye

**Affiliations:** National Center of Meat Quality and Safety Control, Jiangsu Collaborative Innovation Center of Meat, Production and Processing, College of Food Science and Technology, Nanjing Agricultural University, Nanjing 210095, China; changyw0174@163.com (Y.C.); yongkangwang123@163.com (Y.L.); baiyun@njau.edu.cn (Y.B.); tengshuang@njau.edu.cn (S.T.); 17865561759@163.com (Y.G.); douhan1019@163.com (H.D.)

**Keywords:** snowflake beef, deep freezing, quality

## Abstract

In order to elucidate whether deep freezing could maintain the quality of snowflake beef, three different deep freezing temperatures (−18 °C, −40 °C, and −60 °C) were used in order to evaluate the changes in tissue structures, quality characteristics and spoilage indexes, and their comparative effects on the quality of snowflake beef. Compared to samples frozen at −18 °C, those stored at −40 °C and −60 °C took a shorter time to exceed the maximum ice crystallization zone (significantly reduced by 2–6 h). In terms of short-term storage, samples frozen at −40 °C and −60 °C had better tissue structure and lower drip loss rate than those frozen at −18 °C; significant differences between groups in drip loss were observed between −18 °C and −60 °C. Moreover, a better bright red color and lower shear force were maintained at −40 °C and −60 °C, with significant differences in shear force between the −18 °C group and the other two groups on day 60. Although there were significant effects on the inhibition of lipid and protein oxidation at −40 °C and −60 °C; no significant variation was observed between these two groups throughout storage. A similar phenomenon was found in flavor, with 1-pentanol identified as an important potential indicator of flavor change in snowflake beef during storage. This study demonstrated that −40 °C and −60 °C had favorable impacts on the quality maintenance of snowflake beef compared to −18 °C. These findings provide a theoretical basis for effective stability of snowflake beef quality during frozen storage.

## 1. Introduction

In recent years, high-end raw meat has become a favorable choice for consumers. Snowflake beef is a typical representative of high-end raw meat, where small and thin veins and plexus fatty layers resemble marble to form a good appearance [1]. Snowflake beef has a content high in unsaturated fatty acids and low in cholesterol. Moreover, its unique flavor, tenderness, and juicy texture after cooking have promoted its acceptability among most consumers.

China has become the dominant country in the world beef import market [2], where the import volume (2.333 million tons) of beef was 1.1 times higher than that in 2020 (2.118 million tons). Due to the current problems of disordered germplasm resources, low breeding rates, and an imperfect standardization system in China, the production of China’s domestic snowflake beef in high quality is lesser. Therefore, some high-quality snowflake beef is mainly imported. Imported snowflake beef must go through long-distance cold chain logistics transportation. Effectively maintaining snowflake beef quality throughout this process could fulfill the domestic consumer demand for high-end meat. The long-distance transportation of raw meat (including the import transportation of beef) mainly relies on frozen storage [3]. Frozen storage can reduce enzymatic activity, slow the oxidation of lipids and proteins, and inhibit the growth and reproduction of microorganisms, thereby slowing down the rate of meat spoilage [4].

Freezing temperatures used for raw meat storage vary from country to country. For example, the freezing temperatures used in the United States and Canada are lower than −18 °C. The maximum allowable limit is −12 °C in the United Kingdom; Japan uses freezing temperatures lower than −18 °C, while the freezing temperature widely adopted in China is −18 °C to −15 °C [5]. Generally, freezing at temperatures below −18 °C can inhibit the biochemical reactions of enzymes and microbial activities in raw meat to some extent, and prolong the shelf life of meat [6]. However, problems such as protein and lipid oxidation could still cause a reduction in meat quality subjected to frozen storage [7]. Previous studies have been conducted on the effects of different freezing temperatures on meat quality maintenance.

In lamb, Choi et al. [8] indicated that deep freezing (below −50 °C) was effective in maintaining its freshness, with significant differences in drip loss. Kim et al. [9] found that the drip losses in pork neck and chicken leg during frozen storage at −60 °C were significantly lower than those frozen at −18 °C, and that deep freezing (−60 °C) was effective in maintaining meat quality over time. Utrera et al. [10] compared the effects of freezing temperatures (−8 °C, −18 °C, and −80 °C) and concluded that frozen storage had a significant effect on TBARS values in beef patties, with −80 °C having the strongest inhibitory effect on lipid oxidation. Li et al. [11] asserted that lower freezing temperatures could effectively reduce the denaturation of beef protein. Moreover, Lee et al. [12] reported that the shelf life of frozen beef products gradually decreased as the frozen temperature increased (−5 °C, −15 °C, and −23 °C). Due to the special quality of snowflake beef, studying the impact of using different freezing temperatures on the storage of snowflake beef could provide a theoretical basis for efficient maintenance of imported snowflake beef quality. The results of frozen storage studies on other products are not suitable for reference. Therefore, the objective of this study was to investigate different degrees of deep freezing (−18 °C, −40 °C, and −60 °C) on snowflake beef preservation by evaluating changes in tissue structures, quality characteristics, and spoilage indexes. The results can help elucidate whether deep freezing maintains the quality of snowflake beef more effectively. This study would provide the theoretical basis for significantly maintaining the quality of high-end beef, such as snowflake beef, in the cold chain logistics process.

## 2. Materials and Methods

### 2.1. Sample Preparation

A quantity of the high rib from the front end of the long backbone between the 1st rib and the 6th–7th ribs of the spine in 28-month-old bulls was purchased from a beef slaughtering company in China. The postmortem time of these samples was 72 h. Afterwards, they were transported to the laboratory using ice boxes. The samples were trimmed of visible connective and adipose tissues, cut parallel to muscle fibers into equal portions of 300 g (approximately 0.08 m × 0.05 m × 0.04 m), and individually packed in sterile ziploc (polyethylene) bags of 0.025-mm thickness without vacuum treatment.

### 2.2. Frozen Storage Conditions

Fifty-two samples of snowflake beef were taken. Meat samples were randomly and equally placed in three freezers at −18 °C, −40 °C, and −60 °C. Physicochemical analyses were performed in quadruplicate after 0, 30, 60, 90, and 173 days of frozen storage at 4 °C. Before the analysis, meat samples were thawed at 4 °C until their core temperatures reached 0–4 °C.

### 2.3. Freezing Temperature Profile

The core temperatures of meat samples in every freezer were monitored during the freezing process. A temperature probe was inserted into the geometrical center of each sample and connected to a temperature logger. The temperature was recorded in 15 min intervals. Four repetitions were done for each treatment group.

### 2.4. Microstructure

The microstructure of snowflake beef under different freezing conditions was analyzed using HE (hematoxylin-eosin) staining. Samples were fixed in a fixative composed of 4% paraformaldehyde and 96% PBS (phosphate buffer saline, which contained 1.9 mmol/L of sodium dihydrogen phosphate dehydrate and 8.1 mmol/L of disodium hydrogen phosphate dodecahydrate). The blocks were then dehydrated in gradients of ethanol (75%, 85%, and 90% for 2 h; 95% for 1h; and 100% for 30 min twice), alcohol benzene (5–10 min), and xylene (5–10 min twice). Then, dehydrated tissues were immersed in paraffin for 3 h at 65 °C. Subsequently, these tissues were embedded in paraffin using an embedding machine (JB-P5, Wuhan Junjie Electronics Co., Ltd., Wuhan, China). After that, the samples were put into the dehydrator (Donatello DIAPATH, Martinengo, Italy) to be dehydrated and cut into sections perpendicular to the direction of the muscle fibers using a frozen meat slicer. Sections were dyed with hematoxylin and eosin solutions and were examined under a biological microscope (BX41, Olympus, Tokyo, Japan). Images were magnified 400 times for analysis. Image J professional image analysis software was used to calculate the percentage of muscle fiber gap area to total muscle bundle area. Four repetitions were done for each treatment group.

### 2.5. Drip Loss

The drip loss of snowflake beef was determined according to a procedure used by Honikel et al. [13], with some slight modifications. The weight of each sample before storage was recorded. After thawing, every sample was removed from the wrapping and re-weighed after wiping drippings from its surface. The rate of drip loss was calculated using the following equation: X = (W_1_ − W_2_)/W_1_ × 100%, where X was drip loss (%); W_1_ was the weight of meat before storage (g); and W_2_ was the weight of meat after thawing (g). Four repetitions were done for each treatment group.

### 2.6. Color

The color of the samples was measured using a colorimeter (CR-400, Konica Minolta, Tokyo, Japan). The aperture opening size was 8 mm, and light source was D65. The observer angle used was perpendicular to the surface of samples, in order to obtain accurate recording of the values. Five measurements of color values were made on the surface of each sample. The device was calibrated with a white standard plate before measurements by orienting it vertically to the plate. The color values (L*, a*, b*) of the samples were recorded. Four repetitions were done for each treatment group.

### 2.7. Shear Force Measurement

The samples were cut into 1-cm cylindrical samples parallel to the muscle fiber. A texture analyzer (C-LM3B, Northeast Agricultural University, Harbin, China) was used to measure shear force. The maximum force cut into the samples was recorded and expressed in Newtons (N). Four repetitions were done for each treatment group.

### 2.8. Thiobarbituric Acid Reactive Substances (TBARS)

The TBARS of snowflake beef were determined according to the procedure used by Erkan et al. [14], with some slight modifications. Five grams of minced samples were homogenized with 25 mL of 7.5% trichloroacetic acid (containing 0.1% of EDTA). The mixture was centrifuged at 10,000 rpm at 4 °C for 10 min. Two mL of the collected supernatant were mixed with 0.02 mol/L of thiobarbituric acid solution (2 mL) and incubated in water at 95 °C for 30 min. The absorbance of each sample was measured at 532 nm. Using a standard curve of tetraethoxy-propane, the results were determined using malondialdehyde as a standard. The TBARS levels were expressed in mg of malondialdehyde/kg of the minced sample, which was calculated using the following equation: X = (c × V × 1000)/(m × 1000), where X was the content of malondialdehyde in the specimen (mg/kg); c was the concentration of malondialdehyde in the sample solution obtained from the standard series curve (µg/mL); V was the volume of sample solution (mL); and m was the mass of the sample represented by the final sample solution (g). Four repetitions were done for each treatment group.

### 2.9. Carbonyl Content Determination

Carbonyl content was extracted using the test kit (A087-1-2, Jiancheng Institute of Biological Engineering, Nanjing, China). Briefly, as required by the kit, 0.1 mL of supernatant for protein carbonyl determination was taken after the samples were homogenized and centrifuged. A portion of the supernatant was also taken in order to determine the total protein concentration of the homogenate using the Komas Brilliant Blue test kit (A045-1, Nanjing Jiancheng Institute of Biological Engineering, Nanjing, China). The supernatant of the experimental group was mixed with 0.4 mL of Reagent III and incubated at 37 °C in the dark for 30 min, while 0.4 mL of Reagent IV instead of Reagent III was added to the blank sample. The liquid of each group was mixed separately with Reagent V and was vortexed for 1 min. The mixture was centrifuged at 12,000 rpm at 4 °C for 10 min. The precipitate was washed with a 1-mL mixture of ethanol and ethyl acetate (1:1, *v*/*v*) until it turned white. The mixture was vortexed for 1 min and centrifuged at 12,000 rpm at 4 °C for 10 min. The pellets were dissolved in 1.25 mL of Reagent VI, and the mixture was dissolved in a water bath at 37 °C for 15 min. Subsequently, the mixture was vortexed until the entire precipitate had been dissolved, and then centrifuged at 12,000 rpm at 4 °C for 15 min. The carbonyl concentration from the absorbance at 370 nm was calculated using the following equation, which was expressed as nmol per milligram of protein: X = (A_1_ − A_2_)/(22 × d × c) × 125 × 100,000, where X was protein carbonyl content (nmol/mg prot); A_1_ was the absorbance of the experimental group; A_2_ was the absorbance of the control group; d was colorimetric optical diameter (cm); and c was protein concentration of the sample measured by the KOMAS kit (mg prot/L). Four repetitions were done for each treatment group.

### 2.10. Volatile Organic Compounds

The volatile organic compounds (VOCs) of snowflake beef were analyzed by GC-IMS (gas phase ion migration spectrum) based on a previous method by Garrido-Delgado et al. [15], with some slight modifications. Three grams of minced samples were weighted in a 20-mL vial. After 10 min of incubation at 60 °C, 500 μL of sample headspace was automatically injected using a heated syringe (85 °C). After injection, nitrogen gas was used as a carrier gas to transfer the sample into the FS-SE-54-CB-1 column (15 m × 0.53 mm, 1 μm) at 2 mL/min over the first 2 min. Then, the rate was linearly increased up to 15 mL/min during the next 8 min, to 80 mL/min during the next 10 min, to 130 mL/min during the next 15 min, and then to 145 mL/min during the remaining 5 min. The drift tube was operated at a pressure of 1.381 kPa, a temperature of 45 °C, and a drift gas flow rate of 150 mL/min (nitrogen). The temperature of the IMS detector was 45 °C. Two-dimensional GC-IMS data were acquired using LAV (laboratory analytical viewer) software included with the instrument. The GC-IMS library search software with the NIST2014 database and IMS database was used to analyze the characteristic flavor substances qualitatively; the Gallery Plot plug-in in LAV was used to compare the differences of different volatile substances quantitatively. Four repetitions were done for each treatment group.

### 2.11. Statistical Analysis

Four repetitions of each experiment were carried out. The significance of the main effects was determined using the one-way ANOVA procedure of SAS 8.0. The determination of significant differences (*p* < 0.05) among the means was performed by Duncan’s multiple range test. Most of the graphs were generated by using GraphPad Prime 8.

## 3. Results and Discussion

### 3.1. Microstructure

**Figure 1:**
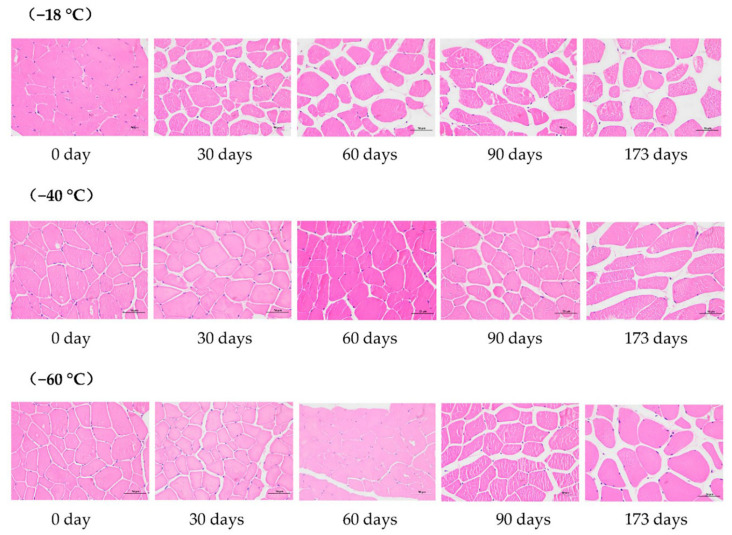
Effects of different freezing storage temperatures on ice crystal formation in snowflake beef (*n* = 4).

As shown in Figure 1, differences in voids between muscle fibers were observed at −18 °C on day 30 when the −40 °C and −60 °C groups did not vary. During the later freezing period, the myofibrillar structures were broken into fragments of different sizes in the −18 °C group. However, −40 °C group had more intact cell morphology and smaller interstices.

**Figure 2:**
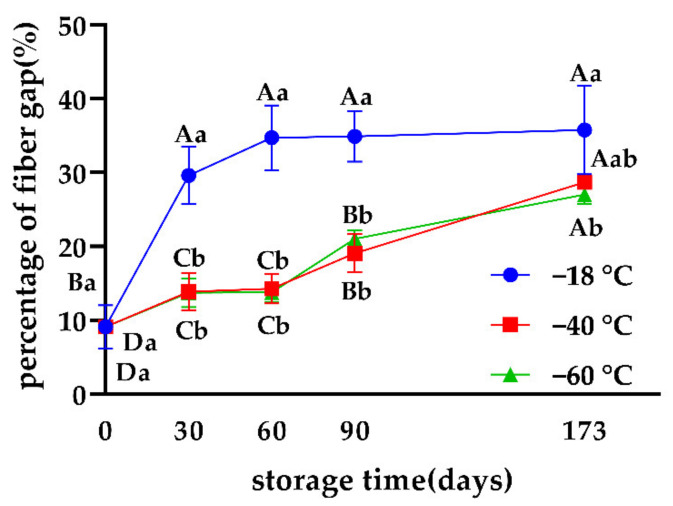
Effects of different freezing storage temperatures on fiber gap percentages of snowflake beef (*n* = 4); ^ab^ represents significant differences of different freezing temperatures for the same storage time; ^ABCD^ represents significant differences of different freezing times for the same storage temperature.

The results in Figure 2 show that after 30 days of freezing, the percentage of space between muscle fibers in the −18 °C group increased sharply, significantly higher than that in the −40 °C and −60 °C groups (*p* < 0.05). This may be due to the creation of larger ice crystals, which formed larger gaps. Results indicated that throughout the freezing process, the muscle fiber structure of the −40 °C and −60 °C groups were consistently more dense than those of the −18 °C treatment, a finding which was consistent with Kaale et al. [16] who reported formation of small crystals at a lower temperature that were evenly distributed both inside and outside the cells, leading to less damage to the tissue. Li et al. [11] confirmed that beef samples stored at lower temperatures were more compact, ensuring better water-holding capacity.

### 3.2. Freezing Temperature Profile and Drip Loss

**Figure 3:**
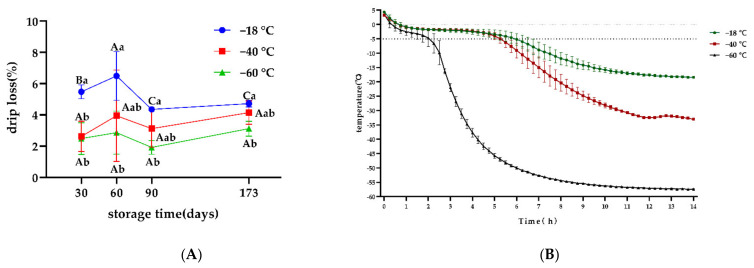
(**A**) Effects of different freezing storage temperatures on drip loss of snowflake beef (*n* = 4).; (**B**) Effects of different freezing storage temperatures on freezing temperature curve of snowflake beef (*n* = 4); ^ab^ represents significant differences of different freezing temperatures for the same storage time; ^ABC^ represents significant differences of different freezing times for the same storage temperature.

Drip loss is an important index used to evaluate the quality of frozen meat after thawing. The rupture of fiber membrane and destruction of muscle tissues was mainly caused by ice crystals, while protein denaturation decreased hydration capacity. The released water was then redistributed into the sarcoplasmic and extracellular spaces, and then drip loss was formed during thawing [3]. As shown in Figure 3A, compared to the samples stored at −18 °C, the advantage of freezing at −40 °C and −60 °C in terms of maintaining the water-holding capacity was observed over the period, where significant differences between −18 °C and −60 °C were observed on days 30, 60, 90, and 173 (*p* < 0.05). This indicated that freezing at −40 °C and −60 °C could significantly reduce drip loss in snowflake beef compared to being frozen at −18 °C (*p* < 0.05), which was similar to the results reported by Kim et al. [9], who indicated that deep freezing was advantageous in maintaining the drip loss. Current studies on the factors that influence the water-holding capacity of frozen meat were mainly focused on the physical effects of ice crystal growth, muscle protein denaturation, and lipid oxidation. As shown in Figure 3B, the time to pass through the maximum ice crystallization zone decreased with a decrease in the freezing temperature. A low freezing rate in the −18 °C group may induce the formation of large and irregular ice crystals, thereby leading to damage to tissue structures of the samples, inducing drip loss [17,18]. This result was consistent with Lee et al. [19], who found that the drip loss of pork tenderloin frozen at −18 °C was higher than that frozen at −50 °C and −60 °C. Moreover, altered hydration caused by protein denaturation may be another reason for affecting the rate of drip loss [20].

Throughout the freezing process in this study, the drip loss of snowflake beef was found to rise initially and then to fall with the freezing time, while the drip loss on day 90 was significantly lower than that on day 60 in all groups (*p* < 0.05). The decrease in the water-holding capacity during short periods may be due to the transformation of water into ice crystals during frozen temperature storage, which may disrupt muscle tissue structure to effect change in muscle fiber space expansion and water migration within the muscle. Musculoskeletal proteins were destroyed, and the ability to bind water was reduced, a finding which was consistent with the results of Lee et al. [19]. After a long duration of storage, the drip loss of snowflake beef was significantly higher (*p* < 0.05), which may be a result of surface dehydration of the meat; the internal water was reabsorbed after migrating to the surface cells. Then, the reabsorption of muscle fibers occurred, resulting in lower drip loss.

### 3.3. Color

**Figure 4:**
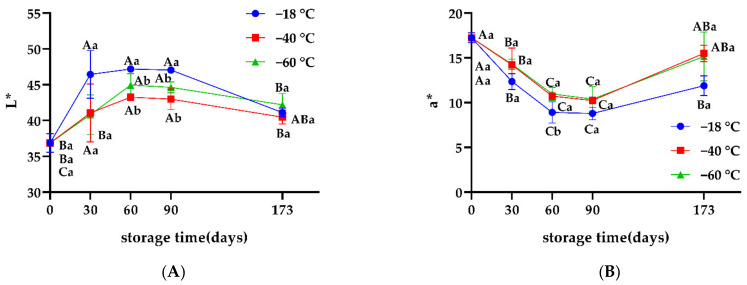
(**A**) Effects of different freezing storage temperatures on L* of snowflake beef (*n* = 4); (**B**) Effects of different freezing storage temperatures on a* of snowflake beef (*n* = 4); ^ab^ represents significant differences of different freezing temperatures for the same storage time; ^ABC^ represents significant differences of different freezing times for the same storage temperature.

The color of meat is an important element in assessing the quality of meat, which also changes during frozen storage. Generally, myoglobin in muscle mainly exists in the form of deoxymyoglobin (DMb), oxymyoglobin (OMb), and methemoglobin (MMb). The chemical properties of myoglobin are key to determining meat color. Before meat muscle is destroyed, most of the myoglobin exists in the form of DMb, and appears dark purple. When the meat is in contact with oxygen, the DMb on the surface is gradually replaced by OMb, which appears bright red. After long-term storage, the myoglobin mainly exists in the form of MMb, which appears brownish. Compared to storage at −18 °C, L* was found to be lower when samples were frozen at −40 °C and −60 °C, which may be attributed to meat being increased with free water released during thawing (Figure 4). The a* in the −40 °C and −60 °C groups was significantly higher than those in the −18 °C group (*p* < 0.05), especially on day 60 when the color of samples frozen at −18 °C changed from their original bright red to dark red, while the color could still maintain a better bright red at −40 °C and −60 °C. This also could be seen intuitively by visual sensory analysis. This result was consistent with the findings of Wang et al. [21], who indicated that the oxidation of oxygenated myoglobin to high iron myoglobin occurred because of the lower freezing temperature.

### 3.4. Shear Force

**Figure 5:**
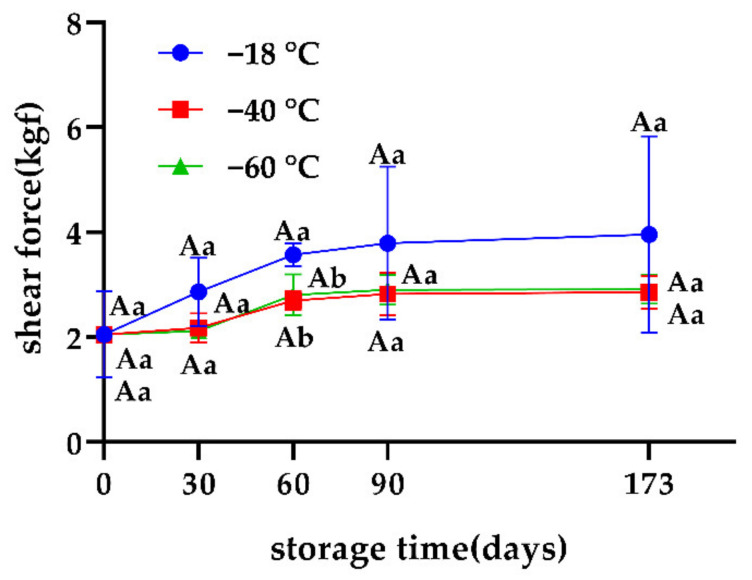
Effects of different freezing storage temperatures on shear force of snowflake beef (*n* = 4); ^ab^ represents significant differences of different freezing temperatures for the same storage time; ^A^ represents significant differences of different freezing times for the same storage temperature.

Tenderness directly reflects the chewiness and overall texture of meat in the mouth, and hence is an important indicator to evaluate the quality of snowflake beef. The highest shear force among the three groups was observed at −18 °C (Figure 5). This phenomenon may be because when the freezing temperature is lower, the ice crystals are more abundant and finer, and the destruction of muscle fiber structure is less, which is supported by Kim et al. [22]. It was concluded that −40 °C and −60 °C could maintain the tenderness of snowflake beef better compared to a freezing temperature of −18 °C.

### 3.5. TBARS and Carbonyl Content

**Figure 6:**
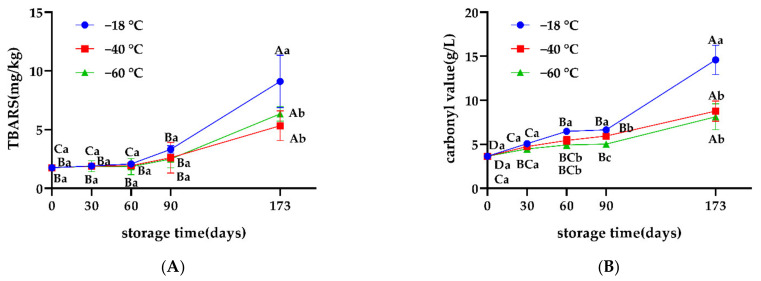
(**A**) Effects of different freezing storage temperatures on TBARS of snowflake beef (*n* = 4); (**B**) Effects of different freezing storage temperatures on carbonyl values of snowflake beef (*n* = 4); ^abc^ represents significant differences of different freezing temperatures for the same storage time; ^ABCD^ represents significant differences of different freezing times for the same storage temperature.

The indexes of TBARS and carbonyl were important markers to reflect the fat oxidation and protein oxidation in meat, respectively. The oxidation of fat and protein are integral precursors for color and flavor variations in meat. Continuous increases in TBARS and carbonyl values were observed in all groups throughout storage, which indicated that lipid and protein oxidation could still occur at freezing temperatures lower than −18 °C (Figure 6). There was a significant correlation between lipid oxidation and protein oxidation during frozen storage, which was supported by the studies of Utrera et al. [10] and Soyer et al. [23], who indicated that primary (hydroperoxides) and secondary (aldehydes) oxidation products of lipids could serve as substrates for protein oxidation [24].

TBARS values in the −40 °C and −60 °C groups were always lower than those in the −18 °C group. The difference was significant on day 173 (*p* < 0.05), which may be because the lower freezing temperature effectively inhibited the oxidation of unsaturated fatty acids during the long-term freezing process, reducing the extent of further lipid oxidation in the meat. This corroborated the results of Wang et al. [21] and Lan et al. [25], who found that the oxidative rancidity of lipids could be effectively reduced by lowering the freezing temperature. Unfrozen water during frozen storage triggers lipid oxidation (peroxidation) in meat, and temperature affects the occurrence of this chemical reaction by influencing the proportion of unfrozen water [3]. Its product, fat hydroperoxide, is correlated with TBARS values in the early stages of storage. In late storage, ice crystals can damage cell membranes and release pro-oxidants, especially heme iron, into the sarcoplasm. Iron is considered a potential factor in the relationship between TBARS and fat content in the later storage period [26,27,28].

After 60 days, the carbonyl values in the −40 °C and −60 °C groups were significantly lower than those in the −18 °C group (*p* < 0.05), which indicated that −40 °C and −60 °C were more favorable for inhibiting the oxidation of proteins in snowflake beef during long periods of freezing. A possible reason for this is that shorter times spent to pass the zone of maximum ice crystal formation at −40 °C and −60 °C result in smaller and more uniform ice crystals; this causes less damage to the tissue structure of the meat, and hence smaller contact area with oxygen to delay protein oxidation. Meanwhile, the lower temperature had a stronger inhibitory effect on enzymatic activity, which may result in less protein oxidation. Jiang et al. [29] confirmed that lower freezing temperatures result in less Ca^2+^-ATPase activity and improved protein integrity. Utrera et al. [10] found that frozen storage significantly affected oxidative changes of specific proteins in meat which were temperature-dependent.

### 3.6. Volatile Organic Compounds

VOCs produced by microbial growth and metabolism are important sources of spoilage odor in meats. Temperature is one of the most important factors affecting the selection and growth of microbes during fresh meat storage. Different temperatures affect the evolution of flora in cooled meat and different types of metabolic activities of bacteria, which ultimately determines the type and content of VOCs released during the spoilage of meat. Notably, 15 VOCs were identified in fresh snowflake beef, including 6 alcohols, 5 aldehydes, 3 ketones, and 1 ester (Table 1). After 30 days of storage, there was no significant variation among the groups for the main volatile components, such as 1-propanol, 1-butanol, 1-pentanol, hexanal, butyraldehyde, 2-heptanone, and ethyl acetate. After 60 days of storage, the relative contents of benzaldehyde, 2-butanone and 2-pentanone in all samples showed different degrees of increase, with benzaldehyde adding a stronger sweet and bitter taste of almond and cherry. This may be due to the concentration of VOCs above the threshold resulting from endogenous enzymes and microbial action that produce an unpleasant odor. At 173 days of storage, the content of 1-butanol and 1-pentanol significantly increased in the −18 °C group compared to the −40 °C and −60 °C groups (*p* < 0.05). Amyl alcohol was reported to be detected in beef in the early stages of spoilage at significantly increased levels [30]. Therefore, in this study, 1-pentanol was identified as a potential spoilage indicator associated with the freshness of snowflake beef, which may be the important compound that caused the difference in odor between −18 °C and others, with the −18 °C group having a less acceptable odor than the −40 °C and −60 °C groups.

## 4. Conclusions

This study demonstrated that the freezing rate of snowflake beef gradually increased with a decline in freezing temperature, and that the time to pass the maximum ice crystallization zone at −40 °C and −60 °C was shorter than that at −18 °C. In terms of short-term storage of snowflake beef, compared to −18 °C, −40 °C and −60 °C could better maintain the structural integrity of snowflake beef, significantly reduce the drip loss of snowflake beef, and better maintain the color and tenderness, thus more effectively maintaining the overall quality of snowflake beef. For a long period of freezing, −40 °C and −60 °C could effectively inhibit the oxidative degradation of protein, the oxidation of lipids, and flavor deterioration in snowflake beef, where 1-pentanol could be used as an important indicator of flavor change in snowflake beef during storage. This study could provide the theoretical basis for the cold chain logistics temperature of international trade for high-end meat.

## Figures and Tables

**Table 1:** Effects of different freezing storage temperatures on volatile organic compounds in snowflake beef (*n* = 4); ^abc^ represents significant differences of different freezing temperatures for the same storage time.

Time	0 Days	30 Days			60 Days			90 Days			173 Days		
Volatile Organic Compounds		−18 °C	−40 °C	−60 °C	−18 °C	−40 °C	−60 °C	−18 °C	−40 °C	−60 °C	−18 °C	−40 °C	−60 °C
Alcohols													
1-Propanol	3150 ± 96	697 ± 77 ^a^	695 ± 17 ^a^	807 ± 107 ^a^	889 ± 57 ^b^	844 ± 40 ^b^	1695 ± 57 ^a^	2872 ± 347 ^a^	3096 ± 153 ^a^	2223 ± 156 ^b^	285 ± 93 ^b^	368 ± 33 ^b^	590 ± 121 ^a^
1-Butanol	526 ± 43	670 ± 114 ^a^	763 ± 54 ^a^	756 ± 33 ^a^	-	-	-	-	-	-	876 ± 12 ^a^	563 ± 69 ^b^	426 ± 98 ^c^
1-Pentanol	829 ± 20	765 ± 68 ^a^	777 ± 17 ^a^	676 ± 128 ^a^	702 ± 20 ^a^	625 ± 17 ^b^	447 ± 56 ^c^	360 ± 74 ^a^	427 ± 67 ^a^	419 ± 68 ^a^	981 ± 7 ^a^	954 ± 15 ^b^	955 ± 10 ^b^
n-Hexanol	581 ± 181	812 ± 3 ^a^	815 ± 5 ^a^	701 ± 110 ^a^	732 ± 111 ^a^	772 ± 7 ^a^	578 ± 132 ^a^	434 ± 60 ^b^	521 ± 74 ^ab^	550 ± 37 ^a^	930 ± 18 ^a^	426 ± 135 ^c^	711 ± 110 ^b^
Heptanol	-	-	-	-	-	-	-	30 ± 5 ^a^	43 ± 18 ^a^	36 ± 10 ^a^	-	-	-
2-Hexen-1-ol	180 ± 37	173 ± 46 ^a^	190 ± 13 ^a^	137 ± 64 ^a^	63 ± 37 ^a^	62 ± 26 ^a^	31 ± 16 ^a^	-	-	-	124 ± 13 ^a^	100 ± 15 ^a^	94 ± 26 ^a^
1-Octen-3-ol	529 ± 110	835 ± 28 ^a^	965 ± 57 ^a^	836 ± 146 ^a^	208 ± 23 ^a^	222 ± 32 ^a^	127 ± 34 ^b^	343 ± 51 ^b^	588 ± 189 ^a^	265 ± 62 ^b^	635 ± 98 ^a^	409 ± 67 ^b^	519 ± 38 ^ab^
2ethyl-1-hexanol	-	63 ± 5 ^a^	62 ± 4 ^a^	55 ± 2 ^b^	158 ± 51 ^a^	194 ± 29 ^a^	119 ± 36 ^a^	-	-	-	-	-	-
Aldehydes													
Butanal	-	-	-	-	-	-	-	1596 ± 338 ^a^	1384 ± 175 ^a^	1720 ± 76 ^a^	-	-	-
Pentanal	769 ± 33	735 ± 37 ^a^	748 ± 11 ^a^	623 ± 109 ^b^	725 ± 19 ^a^	681 ± 24 ^a^	243 ± 84 ^b^	-	-	-	-	-	-
Hexanal	664 ± 24	652 ± 28 ^a^	632 ± 17 ^a^	640 ± 54 ^a^	630 ± 30 ^a^	594 ± 23 ^a^	474 ± 75 ^b^	431 ± 11 ^b^	492 ± 38 ^a^	449 ± 43 ^ab^	859 ± 5 ^a^	897 ± 36 ^a^	881 ± 15 ^a^
Heptanal	553 ± 42	472 ± 63 ^a^	452 ± 57 ^a^	400 ± 85 ^a^	245 ± 63 ^a^	257 ± 78 ^a^	180 ± 65 ^a^	199 ± 69 ^a^	226 ± 19 ^a^	207 ± 18 ^a^	-	-	-
Octanal	245 ± 6	223 ± 14 ^a^	217 ± 12 ^a^	183 ± 22 ^b^	290 ± 39 ^a^	299 ± 21 ^a^	204 ± 77 ^b^	111 ± 29 ^b^	162 ± 16 ^a^	122 ± 12 ^b^	916 ± 93 ^a^	696 ± 116 ^b^	814 ± 18 ^ab^
n-Nonanal	324 ± 9	301 ± 14 ^a^	295 ± 16 ^a^	252 ± 23 ^b^	60 ± 5 ^ab^	67 ± 6 ^a^	46 ± 18 ^b^	193 ± 36 ^b^	273 ± 19 ^a^	254 ± 24 ^a^	237 ± 10 ^a^	209 ± 27 ^a^	229 ± 16 ^a^
Benzaldehyde	-	53 ± 15 ^a^	57 ± 5 ^a^	39 ± 10 ^a^	260 ± 43 ^a^	248 ± 56 ^a^	47 ± 35 ^b^	-	-	-	568 ± 14 ^a^	572 ± 3 ^a^	568 ± 20 ^a^
Ketones													
2-Propanone	-	3147 ± 299 ^b^	3342 ± 105 ^ab^	3655 ± 172 ^a^	3113 ± 52 ^a^	3166 ± 45 ^a^	3187 ± 407 ^a^	-	-	-	-	-	-
2-Butanone	-	332 ± 88 ^a^	406 ± 11 ^a^	441 ± 78 ^a^	589 ± 50 ^a^	628 ± 32 ^a^	404 ± 83 ^b^	2130 ± 488 ^a^	1969 ± 522 ^a^	2404 ± 222 ^a^	-	-	-
2-Pentanone	-	282 ± 59 ^a^	270 ± 35 ^a^	257 ± 57 ^a^	421 ± 48 ^a^	376 ± 69 ^a^	373 ± 71 ^a^	-	-	-	-	-	-
2-Hexanone	-	130 ± 4 ^a^	141 ± 10 ^a^	119 ± 36 ^a^	155 ± 59 ^a^	140 ± 55 ^a^	78 ± 26 ^a^	-	-	-	-	-	-
2-Heptanone	144 ± 19	201 ± 23 ^a^	238 ± 41 ^a^	219 ± 33 ^a^	44 ± 34 ^a^	25 ± 2 ^a^	28 ± 16 ^a^	166 ± 30 ^b^	152 ± 36 ^b^	354 ± 153 ^a^	166 ± 16 ^a^	133 ± 60 ^a^	145 ± 45 ^a^
2-Octanone	-	49 ± 23 ^a^	66 ± 19 ^a^	46 ± 31 ^a^	71 ± 33 ^a^	65 ± 4 ^ab^	26 ± 2 ^b^	-	-	-	-	-	-
Cyclohexanone	-	62 ± 5 ^a^	67 ± 5 ^a^	61 ± 6 ^a^	-	-	-	-	-	-	-	-	-
2,3-Pentanedione	286 ± 42	-	-	-	-	-	-	-	-	-	-	-	-
2,3-Butanedione	402 ± 25	332 ± 120 ^b^	402 ± 60 ^b^	665 ± 112 ^a^	630 ± 71 ^b^	802 ± 68 ^a^	205 ± 93 ^c^	257 ± 64 ^a^	267 ± 70 ^a^	224 ± 26 ^a^	274 ± 20 ^b^	193 ± 94 ^b^	432 ± 36 ^a^
Esters													
Ethylacetate	873 ± 89	794 ± 27 ^a^	732 ± 76 ^a^	742 ± 29 ^a^	437 ± 59 ^a^	513 ± 99 ^a^	431 ± 47 ^a^	2655 ± 302 ^a^	1402 ± 99 ^b^	3081 ± 337 ^a^	383 ± 32 ^b^	536 ± 33 ^a^	538 ± 48 ^a^
Isopropyl acetate	-	373 ± 99 ^b^	588 ± 102 ^a^	499 ± 85 ^ab^	-	-	-	319 ± 52 ^b^	551 ± 97 ^a^	365 ± 22 ^b^	-	-	-
Isoamyl acetate	-	-	-	-	-	-	-	31 ± 5 ^a^	34 ± 5 ^a^	60 ± 34 ^a^	-	-	-
Others													
Styrene	-	-	-	-	-	-	-	117 ± 48 ^a^	128 ± 15 ^a^	94 ± 7 ^a^	77 ± 3 ^a^	45 ± 10 ^b^	45 ± 6 ^b^
Methylpyrazine	-	101 ± 55	123 ± 24 ^a^	119 ± 42 ^a^	-	-	-	-	-	-	-	-	-
Acetophenone	-	-	-	-	-	-	-	-	-	-	205 ± 1 ^a^	205 ± 3 ^a^	209 ± 7 ^a^

## Data Availability

Data are contained within the article.
